# Z-scheme overall water splitting on photocatalyst sheet mediated by carbon nanotubes using oxysulfide photocatalyst responsive to long wavelengths

**DOI:** 10.1039/d5sc05277g

**Published:** 2025-09-23

**Authors:** Long Wang, Chen Gu, Tsuyoshi Takata, Nobuyuki Zettsu, Swapnil S. Karade, Swarnava Nandy, Joji Yoshimura, Yasutaka Nishi, Kiyoshi Kanie, Takashi Hisatomi, Kazunari Domen

**Affiliations:** a Institute for Aqua Regeneration, Shinshu University Wakasato 4-17-1 Nagano-shi Nagano 380-8553 Japan; b School of Materials and Energy, Guangdong University of Technology Guangzhou 510006 China; c Research Initiative for Supra-Materials, Shinshu University Wakasato 4-17-1 Nagano-shi Nagano 380-8553 Japan; d Nikon Corporation 10-1, Asamizodai, 1-chome, Minami-ku Sagamihara-City 252-0328 Japan; e International Center for Synchrotron Radiation Innovation Smart, Tohoku University Sendai Miyagi 980-8577 Japan; f Office of University Professors, The University of Tokyo 2-11-16 Yayoi Bunkyo-ku Tokyo 113-8656 Japan domen@chemsys.t.u-tokyo.ac.jp

## Abstract

Narrow-bandgap photocatalysts enable broad visible-light absorption and, theoretically, should provide high solar-to-hydrogen (STH) energy conversion efficiencies. However, the actual performance of such materials is highly dependent on the design and fabrication of the water-splitting system. In this study, photocatalyst sheets were constructed using Ga-doped La_5_Ti_2_Cu_0.9_Ag_0.1_O_7_S_5_ (Ga-LTCA, with absorption at *λ* < 700 nm) as the hydrogen evolution photocatalyst together with BiVO_4_ (BVO) as the oxygen evolution photocatalyst, and carbon nanotubes (CNTs) as the electron mediator, employing a cost-effective filtration process. The three components were tightly integrated on a filter paper substrate, allowing the resulting sheets to drive Z-scheme overall water splitting (OWS). Using a two-step Cr_2_O_3_ deposition to coat Ga-LTCA/CNTs/BVO sheets was found to prolong the stability of the system at elevated background pressures. Loading of tin-doped indium oxide (ITO) nanoparticles also enhanced the performance of BVO, and increased the Z-scheme OWS activity. The fabricated sheet exhibited an optimal STH efficiency of 0.17% during Z-scheme OWS reaction in pure water without stirring. This work demonstrates the rational assembly of photocatalyst sheet systems incorporating a long-wavelength-responsive oxysulfide photocatalyst with low-cost carbon materials-based mediators and superior co-catalysts loading strategy, highlighting the potential for scalable hydrogen production *via* photocatalytic OWS.

## Abstract 2

A Z-scheme photocatalyst sheet prepared by filtration offers new opportunities for efficient, scalable production of hydrogen using narrow-bandgap photocatalysts.

## Graphical abstract

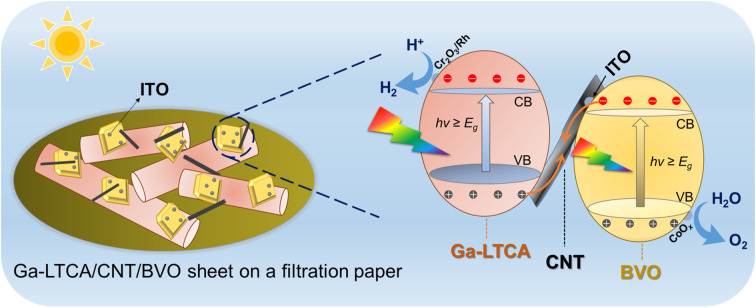

## Introduction

Photocatalytic overall water splitting (OWS) mediated by particulate semiconductors has emerged as a frontier technology for sustainable hydrogen production through direct solar energy conversion.^1–9^ Our research group recently demonstrated a 100 m^2^ photocatalyst panel array capable of scalable, stable, long-term hydrogen and oxygen evolution under ambient conditions.^10,11^ Nevertheless, this system employed Al-doped SrTiO_3_ as the photocatalyst material, which has a wide bandgap of approximately 3.2 eV and absorbs only a limited portion of the solar spectrum.^12,13^ The development of narrow-bandgap photocatalysts is an important aspect of improving the solar-to-hydrogen (STH) energy conversion efficiency of such systems based on utilizing visible light.^14–20^ In this context, oxysulfide photocatalysts such as Y_2_Ti_2_O_5_S_2_, Sm_2_Ti_2_S_2_O_5_, and Gd_2_Ti_2_O_5_S_2_, which have suitable bandgap configurations that enable broad visible light absorption while maintaining robust photostability for water splitting, have aroused huge attention among researchers.^21–26^

The two-step photoexcitation (also known as Z-scheme) concept has been examined as an alternative to the one-step excitation OWS process, which imposes stringent requirements on both the reduction and oxidation reactions.^14,27–31^ The former type of system involves the use of a hydrogen evolution photocatalyst (HEP), an oxygen evolution photocatalyst (OEP), and an suitable electron mediator (either a redox couple or a conductive material).^32,33^ Notably, a Z-scheme system requires that the photocatalysts promote only the respective half-reactions, thereby broadening the range of applicable photocatalysts. Our research group has previously developed La_5_Ti_2_Cu_0.9_Ag_0.1_O_7_S_5_ (LTCA) as a promising oxysulfide photocatalyst, exhibiting an absorption edge of approximately 1.8 eV (equivalent to 700 nm).^34^ This material has been widely employed as the HEP in Z-scheme OWS systems. As an example, Ga-doped LTCA was utilized as the HEP in combination with Au and Mo-doped BiVO_4_ (BVO) to fabricate Z-scheme photocatalyst sheet system. After loading a Rh/Cr_2_O_3_-CoO_*x*_ co-catalyst combination, this photocatalyst sheet demonstrated an apparent quantum yield (AQY) of 11.8% at 420 nm and an STH energy conversion efficiency of 0.4% at 4 kPa and 301 K. This performance exceeded most reported photocatalytic OWS systems capable of harvesting the solar spectrum beyond 600 nm.^35^ In another study, a combination of Al and Mg co-doped LTCA and Mo-doped BVO, together with Au as the electron mediator achieved an AQY of 16.3% at 420 nm and an STH efficiency of 0.67% at 4 kPa and 301 K.^36^ These findings highlight the remarkable potential of photocatalyst sheets employing narrow-bandgap oxysulfide HEPs when employed in Z-scheme OWS systems. Even so, the fabrication of such sheet systems is presently time-consuming and involves elaborate, multiple-step particle transfer processes that make scale-up for practical operational conditions challenging. The use of the expensive metal Au as a conductive layer also presents a significant economic challenge when large-scale applications are considered.

Carbon-based conductors, such as graphite, reduced graphene oxide, and carbon nanotubes (CNTs), show superior electron conductivity, cost-effectiveness, and excellent chemical stability.^37–40^ CNTs have been employed as efficient solid-state electron mediators in a system that combines Cr_2_O_3_/Pt with IrO_2_-loaded Sm_2_Ti_2_O_5_S_2_ as the oxysulfide HEP with absorption extending to 600 nm and CoO_*x*_-loaded BVO as the OEP.^32^ This Z-scheme suspension achieved an STH value of 0.15% under near-ambient conditions, although stirring was required to maintain suitable dispersion of the photocatalyst. In contrast to such conventional suspension-based systems, a sheet structure enables integration of the HEP, OEP, and conductor into a single unit. This eliminates the need for stirring and for redox mediators such as Fe^2+^/Fe^3+^ and I^−^/IO_3_^−^, thereby facilitating scale-up under realistic operational conditions. For example, our recent work demonstrated that Sm_2_Ti_2_O_5_S_2_ could be immobilized along with BVO and CNTs on filter paper using a simple and scalable method to form a Z-scheme OWS system with an STH efficiency of 0.22% at 288 K under near-ambient pressure without stirring.^41^ On this basis, it was anticipated that combining an oxysulfide HEP responsive to longer wavelengths with CNTs acting as the electron mediator could provide an effective Z-scheme OWS system.^42^

In the present work, Ga-doped LTCA (Ga-LTCA) was employed as the HEP together with BVO loaded with tin doped indium oxide (ITO) nanoparticles (NPs) as a highly active OEP and CNT as electron transfer mediator. By optimizing the cocatalyst loadings onto the HEP and OEP, a Cr_2_O_3_/Rh/Ga-LTCA-CNT-CoO_*x*_/ITO/BVO photocatalyst sheet for Z-scheme OWS was constructed. This sheet provided an STH energy conversion efficiency of 0.17% at 313 K and 10 kPa. The importance of effective surface modifications of the photocatalyst sheet is a particular focus of this work. This system highlights the considerable potential of employing CNTs to function as cost-effective conductors and demonstrates an economically viable approach to the scaling up of sheet-based systems using long-wavelength-responsive photocatalysts.

## Results and discussion

Photocatalyst sheets were fabricated through a filtration-assisted assembly process, employing Ga-LTCA as the HEP, BVO as the OEP, and CNTs as the conductive electron mediator. Building on our previous work with LTCA-based oxysulfide semiconductors showing enhanced photocatalytic performance, this study used Ga^3+^ doping strategy to optimize charge carrier dynamics in the catalyst. Prior work has indicated that the controlled substitution of Ga into Ti sites significantly enhanced charge separation efficiency. This doping leads to increased sacrificial hydrogen evolution rates compared with those obtained from undoped LTCA.^35^ The Ga-LTCA used in the present research was synthesized *via* a conventional solid-state synthesis and the structure and quality of the material were confirmed through a comparative X-ray diffraction (XRD) analysis in Fig. S1a. Both the original LTCA and Ga-LTCA produced almost identical patterns, indicating that the incorporation of Ga at 1.0 mol% preserved the host crystal structure. In addition, Ga 3d X-ray photoelectron spectroscopy (XPS) results suggested that Ga was presented in the Ga-LTCA (Fig. S1b). Scanning electron microscopy (SEM) observations (Fig. S2) also established that Ga doping maintained the original rod-like morphology of the materials, which comprised rods 5 to 8 μm in length, without inducing particle aggregation or phase segregation. BVO was synthesized using a hydrothermal process and the XRD pattern in Fig. S3 confirms that a polyhedral monoclinic phase of BVO particles (JCPDS no.75-2480) was obtained.

**Fig. 1:**
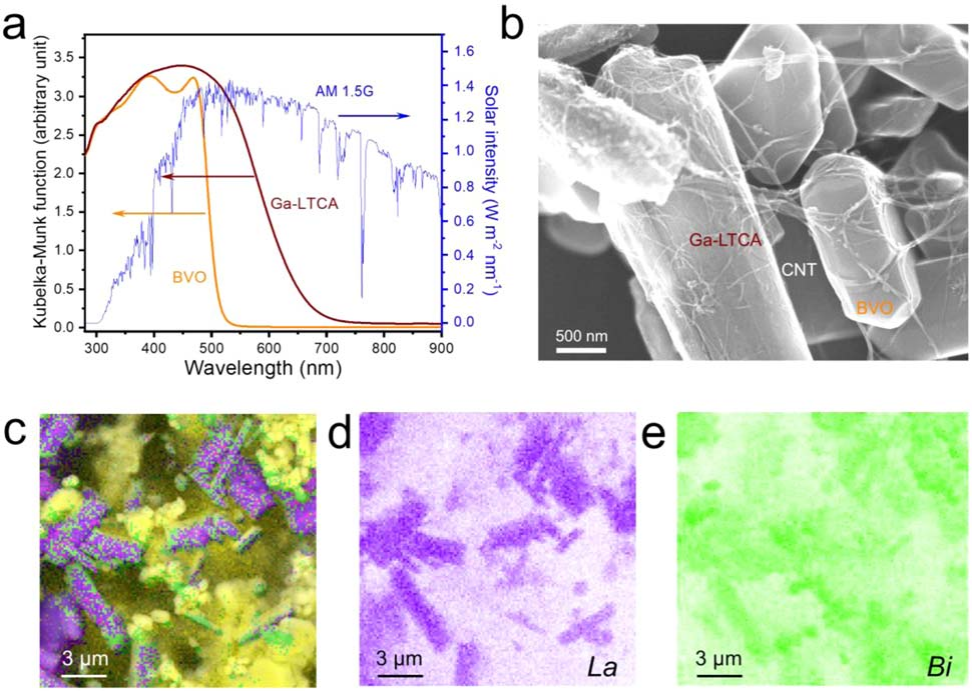
Characterization of Ga-LTCA, BVO, and Ga-LTCA-CNT-BVO samples. (a) UV-vis DRS spectra of Ga-LTCA, BVO, and AM 1.5G. (b) SEM image of Ga-LTCA-CNT-BVO. (c–e) SEM-EDX mapping images of (c) all elements, (d) La, and (e) Bi.

Optical characterization by UV-vis diffuse reflectance spectroscopy (DRS) was used to assess the light-harvesting properties of the photocatalysts (Fig. 1b). The Ga-LTCA exhibits a pronounced absorption edge at 700 nm (equivalent to a bandgap of approximately 1.8 eV), while the BVO showed absorption at shorter wavelengths, terminating at 520 nm (equivalent to a bandgap of 2.4 eV). This difference in the absorption spectra would be expected to lead to complementary light harvesting across a wide range of visible wavelengths from 400 to 800 nm, covering 58% of the air mass 1.5 global (AM 1.5G) solar radiation spectrum. Morphological integration of the components was verified through SEM observations and elemental mapping (Fig. 1c–g). The rod-shaped Ga-LTCA crystals (reflected in the La signal) and BVO crystals (shown by the Bi signal) were evidently interconnected *via* CNTs networks acting as solid conductive mediators, forming continuous charge transport pathways as indicated by the principle of Z-scheme mechanism.

The water-splitting half-reaction activities of Cr_2_O_3_/Rh/Ga-LTCA and CoO_*x*_/BVO photocatalysts were initially investigated in the presence of sacrificial agents to elucidate their photocatalytic performances. The hydrogen evolution activity of Ga-LTCA was assessed using Na_2_S/Na_2_SO_3_ as the hole scavenger under visible light irradiation. Notably, the optimal Rh loading was determined to be 0.4 wt% and the impregnation–reduction method demonstrated superior performance over photodeposition (Fig. S4). This result is consistent with our earlier work and can be attributed to the complete reduction of Rh sites in the case of impregnation–reduction method.^34^ Various cocatalysts were evaluated, including Pt, Ru, Ir, and Pd, and Rh exhibited the highest activity (Fig. S5). The activity also changed as the amount of Ga-LTCA was varied from 50 mg to 300 mg. As shown in Fig. S6, the photocatalytic activity increased as the photocatalyst amount was increased from 50 mg to 200 mg, reaching a maximum of approximately 1.6 mmol h^−1^. However, further increases in photocatalyst loading did not enhance activity, indicating a trade-off between light absorption and transmission losses. The slight decrease in the activity seen with increasing amounts of the photocatalyst may be due to the scattering of the incident light. Fig. S6 shows that the AQY values were correlated with the light absorption curve. The Cr_2_O_3_/Rh/Ga-LTCA demonstrated a peak AQY of 11.5% at 420 nm, comparable to published results. The oxygen evolution activity of BVO was evaluated and is plotted in Fig. S7 for varying masses of this compound. The corresponding AQY values were 25.1% and 20.9% at 420 and 460 nm, respectively (Fig. S7). These experiments confirmed that the individual photocatalyst were capable of reaching 10% or more in the visible light region. Hence, the potential feasibility of efficient Z-scheme OWS using these photocatalysts bridged by CNT acting as the electron mediator was promising to establish.

Cocatalyst loading plays a pivotal role in water-splitting reactions and the cocatalyst loading procedures are known to affect activity. During the fabrication of CNTs-mediated photocatalyst sheets in the present work, the cocatalyst loading sequence was systematically investigated, including Rh and Cr_2_O_3_ on the Ga-LTCA, and CoO_*x*_ on the BVO, while CNT were introduced into the solution prior to sheet filtration without any additional pre-treatment. Table 1 summarizes the photocatalytic OWS activities corresponding to different cocatalyst loading sequences. Entries 1–4 reveal that suboptimal performance (H_2_ evolution rates below 10 μmol h^−1^) was obtained in the absence of Cr_2_O_3_ loading. Comparing entries 5 and 6 indicates that pre-loading CoO_*x*_ onto BVO enhanced the OWS process. Similarly, entries 6 and 10 demonstrate that pre-loading of Rh onto the Ga-LTCA resulted in superior performance, achieving H_2_ and O_2_ evolution rates of 22.6 and 10.1 μmol h^−1^, respectively, compared with the post-loading of Rh (H_2_ and O_2_ rates of 11.2 and 4.7 μmol h^−1^, respectively). These findings underscore the critical importance of pre-loading the Rh and Co cocatalysts. Moreover, pre-loading of Cr_2_O_3_ onto Ga-LTCA enhanced the activity of the photocatalyst sheet, yielding the highest activity among the various trials (entry 8), with H_2_ and O_2_ evolution rates of 110.5 and 52.5 μmol h^−1^, respectively. In contrast, post-loading Cr_2_O_3_ onto the sheet gave reduced performance with revolution rates of 88.7 μmol h^−1^ for H_2_ and 39.9 μmol h^−1^ for O_2_ evolution (entry 9). As shown in Fig. S8, the amount of Cr pre-loaded was optimized, and the 0.9 wt% of Cr displayed the best activity. Post-loading of CoO_*x*_ on the BVO reduced the Z-scheme OWS activity of the sheet significantly regardless of the manner in which the Cr_2_O_3_ was loaded, as indicated by comparing entries 2 and 7, 7 and 8, and 7 and 10. These results collectively indicate that the pre-loading of cocatalysts Rh, Cr_2_O_3_, and CoO_*x*_ greatly enhances the Z-scheme OWS reaction. XPS analyses were conducted to examine the chemical states of the cocatalysts. As shown in Fig. S10, the Rh 3d, Cr 2p, and Co 2p spectra confirmed the successful deposition of these cocatalysts on the photocatalyst surfaces.

**Table 1:** Z-scheme OWS activities of photocatalyst sheets made using different cocatalyst loading sequences. Reaction conditions: distilled water (40 mL), Xe lamp with a cutoff filter (L42), background pressure: 4 kPa

Entry	Rh	Cr_2_O_3_	CoO_*x*_	Z-scheme OWS activities	
				H_2_ (μmol h^−1^)	O_2_ (μmol h^−1^)
1	pre^a^	—	pre^e^	8.3	3.9
2	pre^a^	—	post^f^	7.1	3.4
3	post^b^	—	pre^e^	6.5	3.1
4	post^b^	—	post^f^	5.9	2.8
5	post^b^	post^d^	pre^e^	25.7	11.9
6	post^b^	post^d^	post^f^	11.2	4.7
7	pre^a^	pre^c^	post^f^	34.3	15.8
8	pre^a^	pre^c^	pre^e^	110.5	52.5
9	pre^a^	post^d^	pre^e^	88.7	39.9
10	pre^a^	post^d^	post^f^	22.3	10.1

a Rh was loaded onto the Ga-LTCA before sheet fabrication.

b Rh was loaded onto the photocatalyst sheet using an *in situ* photodeposition method.

c Cr_2_O_3_ was loaded onto the Ga-LTCA before sheet fabrication.

d Cr_2_O_3_ was loaded onto the photocatalyst sheet using an *in situ* photodeposition method.

e CoO_*x*_ was loaded onto the BVO before sheet fabrication.

f CoO_*x*_ was loaded onto the photocatalyst sheet using an *in situ* photodeposition method.

Building upon the optimized cocatalyst loading sequence, the effect of CNT content on OWS activity was investigated. As shown in Fig. S11, a physical mixture of Ga-LTCA and BVO without CNTs exhibited low activity (H_2_: 12.6 μmol h^−1^, O_2_: 5.4 μmol h^−1^). The incremental addition of CNTs led to enhanced activity, reaching a peak at a CNTs massed-based proportion of 1.0 wt% relative to the amount of BVO. Interestingly, an excess of CNTs, lowered the activity, presumably as a consequence of parasitic light absorption. The effects of mass of Ga-LTCA and BVO was also assessed. Optimal activity was observed when using 15 mg of Ga-LTCA and 45 mg of BVO, which was attributed to the uniform distribution of photocatalyst on the filtration paper (Fig. S12). Deviations from these masses adversely affected the activity. A mass ratio of 1 : 3 between the two photocatalysts provided the highest activity, possibly by enhancing the powder distribution or structure stability of the materials. Lower photocatalyst masses reduced the OWS activities based on less homogeneous distributions of the photocatalysts. In addition, higher amounts of the Ga-LTCA and BVO did not provide greater performance, primarily because the backward reaction under darkness was enhanced.

**Fig. 2:**
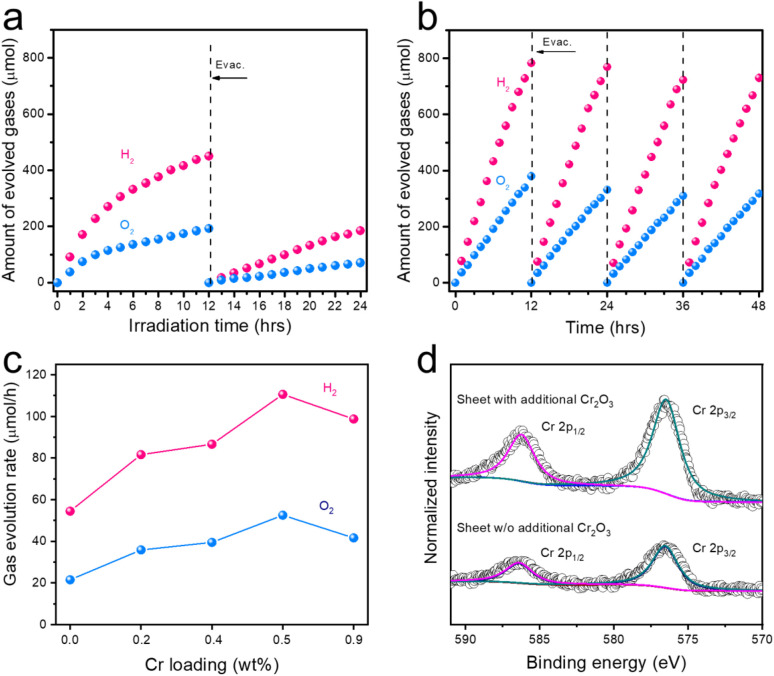
Photocatalytic Z-scheme OWS activity of Cr_2_O_3_/Rh/Ga-LTCA-CNT-CoO_*x*_/BVO photocatalyst sheets. (a) Without and (b) with additional Cr_2_O_3_ loading. (c) Z-scheme OWS activity of Cr_2_O_3_/Rh/Ga-LTCA-CNT-CoO_*x*_/BVO photocatalyst sheets with various amount of additional Cr_2_O_3_, and (d) Cr 2p XPS spectra of photocatalyst with or without additional Cr_2_O_3_ loading.

The photostability of photocatalyst sheets fabricated with the optimized cocatalyst and CNT amounts is an important factor and so was evaluated. As shown in Fig. 2a, the rates of H_2_ and O_2_ evolution gradually declined during a prolonged reaction. In addition, the original activity could not be fully recovered based on evacuation of the system prior to a second run OWS trial, indicating that the diminished activity was largely due to the dissolution of Cr_2_O_3_, as proved by the comparison of Cr XPS in Fig. S9. The lack of an effective surface coating on the Cr_2_O_3_ greatly accelerated the backward reaction (specifically, the oxygen reduction reaction), which was facilitated by active metal sites on the surface that promoted the recombination of H_2_ and O_2_ into water. To address this issue, the sheet was further modified with an additional Cr source (K_2_CrO_4_) by photodeposition in water. As shown in Fig. 2b, the modified photocatalyst sheet demonstrated considerable stability over 12 h of visible light irradiation using a Xe lamp. This improved stability was mainly ascribed to the application of Cr_2_O_3_ coating to the sheet, which inhibited the backward reaction. Optimization of the additional Cr loading based on varying the Cr amount relative to the Ga-LTCA mass in the photocatalyst sheet revealed that 0.5 wt% Cr yielded the highest OWS activity (Fig. 2c). This finding was reinforced by the Cr 2p spectra provided in Fig. 2d. These spectra confirmed that the sheet with additional Cr_2_O_3_ produced more intense Cr 2p_3/2_ and Cr 2p_1/2_ peaks at 575.9 and 586.1 eV, respectively. Subsequently, the additional Cr content was adjusted by varying the K_2_CrO_4_ addition amount from 0.25% to 0.75% as Cr relative to the Ga-LTCA mass. The results, presented in Fig. S13, reveals that an additional loading of 0.5% Cr achieved the best balance between stability and activity. Employing this strategy, cycling experiments were conducted to assess long-term stability. The OWS activities retained 93% of its initial value after four cycles of 12 h reaction (Fig. 2b). These findings demonstrate that the present photocatalyst sheet, formed on filtration paper, exhibits excellent stability, and could potentially be scaled up to larger areas for practical hydrogen production applications.

**Fig. 3:**
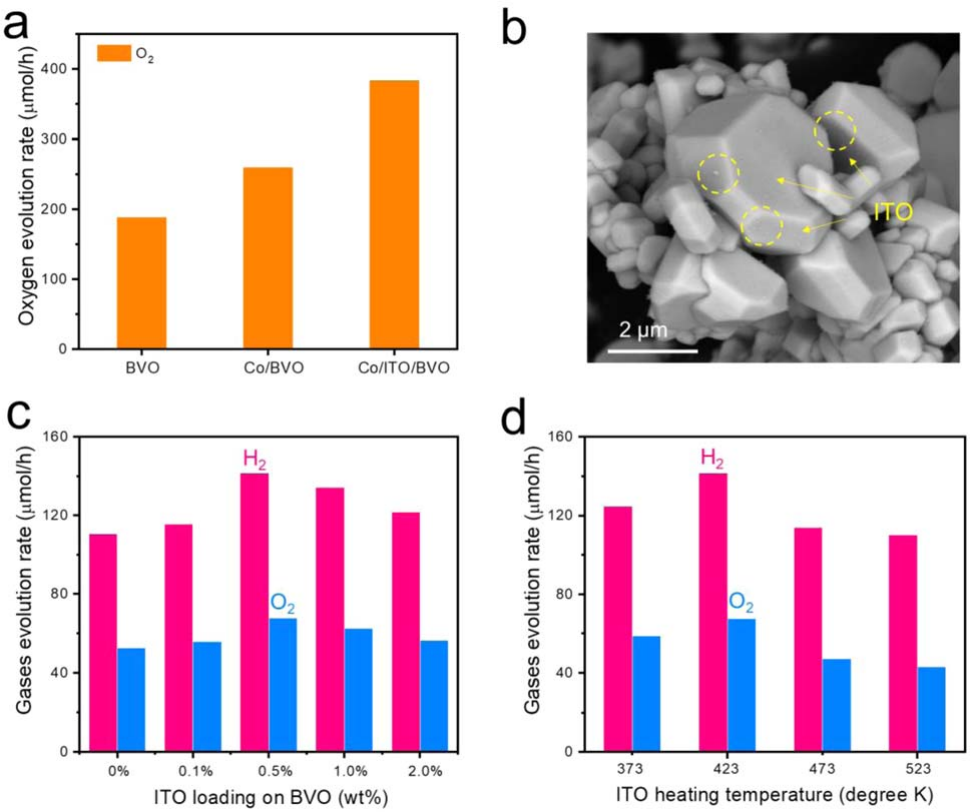
(a) Photocatalytic oxygen evolution activities of BVO with different modifications. (b) SEM image of ITO-loaded BVO. Photocatalytic OWS activity of Cr_2_O_3_/Rh/Ga-LTCA-CNT-CoO_*x*_/ITO/BVO sheet fabricated with (c) different ITO loading amounts and (d) different heating temperatures during ITO loading. Reaction conditions: distilled water (40 mL), Xe lamp with a cutoff filter (L42), background pressure: 4 kPa.

Apart from the modifications of cocatalysts on Ga-LTCA to balance the activity and durability, means of improvement of the activity of BVO were also investigated. It is known that the Co species could increase the oxygen evolution rate by accelerating accumulation and transfer of holes. Hence, the present work modified the BVO with conductive materials to accelerate electron transport. Since the ITO has been reported as an effective conductive mediator for water splitting reaction,^43^ the effect of modifying BVO with ITO NPs was evaluated. Protrusion-rich ITO NPs were chosen here because this material show high water-dispersity as their primary particle state have low resistivity (∼10^−3^ Ω cm) and high transparency in the visible light region (>98%).^44^ These properties were expected to improve their photocatalytic activity under visible light irradiation. Fig. 3a summarizes the oxygen evolution rates obtained using pristine BVO, CoO_*x*_/BVO, and CoO_*x*_/ITO/BVO. The activity of CoO_*x*_/ITO/BVO reached approximately 398 μmol h^−1^ in the presence of AgNO_3_ as the sacrificial reagent and promised a much higher AQY value at 420 nm, higher than the CoO_*x*_/BVO (260 μmol h^−1^) and pristine BVO (180 μmol h^−1^), as shown in Table S1. The ITO NPs were loaded on BVO by simple impregnation and heating method, and were found to be irregularly distributed over the surface of BVO (Fig. 3b and S14). By XPS analysis (Fig. S15), the existence and surface states of Sn and in ITO was confirmed. Accordingly, the effects of the amount of ITO NPs loaded and heat treatment on the OWS performance of the photocatalyst sheet were also investigated. As shown in Fig. 3c, 0.5 wt% of ITO loading greatly improved the OWS activity when using optimized Cr_2_O_3_/Rh/Ga-LTCA-CNT-CoO_*x*_/ITO/BVO photocatalyst sheet, achieving H_2_ and O_2_ evolution rates of 138 and 66 μmol h^−1^, respectively. These values represent an enhancement of 128% compared with a sheet system not including ITO (Table 1, entry 8). The improved activity was mainly because ITO NPs promoted the capture and transfer of electrons from BVO. However, loading with an excess of ITO did not improve the activities, possibly because the ITO NPs occupied too many active sites and thus impeded the loading of CoO_*x*_. Processing at various heating temperatures and under different atmosphere (in N_2_, N_2_ and H_2_, and in air) showed that the highest oxygen evolution rate was achieved under H_2_/N_2_ mixed gases, as shown in Fig. S16, and the optimized heating temperature was at 423 K (Fig. 3d). At higher temperature, the ITO NPs might undergo partly reduction due to the presence of H_2_ atmosphere. These results confirmed that ITO NPs deposited on the BVO were beneficial for capturing and transferring electrons to the substrate, as proved by our former study.^45^ These effects enhanced the half reaction as well as Z-scheme OWS activities. It should be noted that materials that act as electron transfer agents are not limited to ITO NPs. Finding more efficient, low-cost electron transfer agents is likely to enable more efficient hydrogen production by this type of Z-scheme system.

**Fig. 4:**
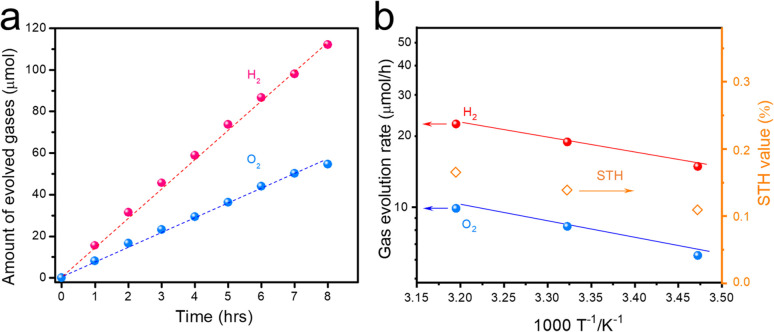
Stability and efficiency of a photocatalyst sheet applied to Z-scheme OWS. (a) Time courses of gas evolution amount over a Cr_2_O_3_/Rh/Ga-LTCA-CNT-CoO_*x*_/ITO/BVO sheet under simulated sunlight (AM 1.5G), and (b) temperature dependence of OWS activity and STH conversion efficiency under simulated sunlight (AM 1.5G). Reaction conditions: distilled water (40 mL), irradiation area: 9 cm^2^.

The activity of the Cr_2_O_3_/Rh/Ga-LTCA-CNT-CoO_*x*_/ITO/BVO photocatalyst sheet was assessed under irradiation from a solar simulator (AM 1.5G) to determine the STH conversion efficiency. As shown in Fig. 4a, the photocatalyst sheet exhibited stable hydrogen and oxygen evolution rates of about 16 and 8 μmol h^−1^, respectively, over a continuous 8 hours light irradiation under conditions of 4 kPa and 288 K. Consequently, the sheet demonstrated an STH efficiency of 0.11%. Additionally, as shown in Fig. 4b, the activity progressively increased with temperature elevation from 288 to 313 K. Correspondingly, the STH values exhibited a similar upward trend, reaching 0.17% at 313 K and 10 kPa. The apparent activation energy of this sheet was estimated to be 13 kJ mol^−1^, aligning with reported values.^46^

**Fig. 5:**
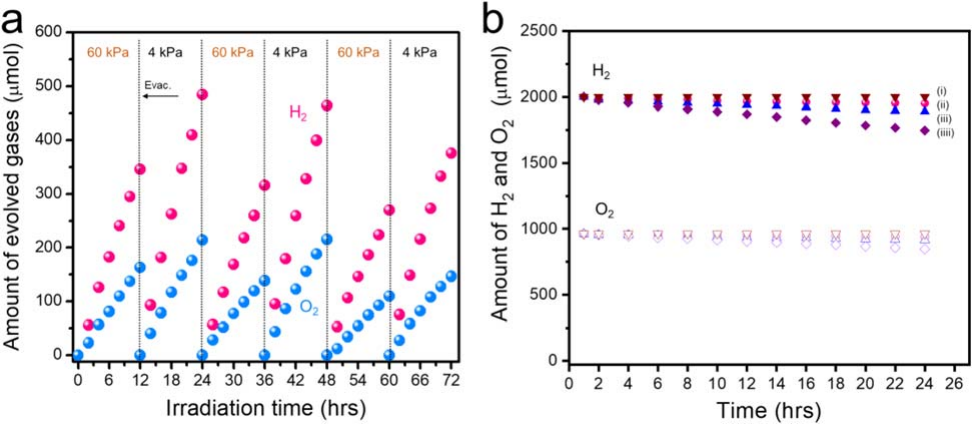
Data from trials assessing pressure-dependent OWS activity and backward reaction progression over Cr_2_O_3_/Rh/Ga-LTCA-CNT-CoO_*x*_/ITO/BVO sheet. (a) Evolution of gases from the Z-scheme system over time under Ar at 60 and 4 kPa in response to irradiation with Xe light (*λ* > 420 nm), and (b) comparison of water formation reaction from gaseous H_2_ and O_2_ using (i) Cr_2_O_3_/Rh/Ga-LTCA-CNT-CoO_*x*_/ITO/BVO sheet, (ii) Cr_2_O_3_/Rh/Ga-LTCA-CNT-CoO_*x*_/BVO sheet, (iii) Rh/Ga-LTCA-CNT-CoO_*x*_/ITO/BVO sheet, (iiii) Rh/Ga-LTCA-CNT-CoO_*x*_/BVO sheet.

Considering the known effect of pressure issues on photocatalytic OWS reactions,^36,41,47^ pressure-dependent activity assessments were performed. As shown in Fig. 5a, during an initial 12 h irradiation trial, an argon (Ar) pressure of 60 kPa resulted in a H_2_ evolution rate of approximately 39 μmol h^−1^. Adjusting the Ar pressure to 4 kPa after the complete evacuation of gases increased the activity to 50 μmol h^−1^. Upon alternating the pressure between 60 kPa and 4 kPa over several cycles, 78% and 87% of the initial activities were retained, respectively. It is evident that the background pressure significantly affects sheet activity, with higher pressures such as 60 kPa leading to an activity loss. This occurred because the backward reaction became more likely as bubbles of H_2_ and O_2_ were formed on and adhered to the sheet surface. A further increase in the background pressure to 90 kPa upon Cr_2_O_3_/Rh/Ga-LTCA-CNT-CoO_*x*_/ITO/BVO was evaluated. As shown in Fig. S17, the OWS activity at 90 kPa was maintained at 77% of that at 60 kPa, demonstrating the effectiveness of the additional Cr_2_O_3_ deposition in preventing the back reaction. The OWS activity of this system was also evaluated under atmospheric pressure in a panel reactor.^41^ In this trial, approximately 4 mL of a mixture of H_2_ and O_2_ gases was evolved during 200 min of exposure to visible irradiation with a Xe lamp, albeit with a gradual decline in the gas evolution rate (Fig. S18). This result confirmed that it was possible to split water to produce H_2_ and O_2_ under ambient condition using this technology. In addition, a mixture of H_2_ and O_2_ was introduced into the reactor to further analyse the vital role of Cr_2_O_3_ layer in preventing the reverse reaction. The consumption of these gases *via* water formation was examined. As shown in Fig. 5b, water was generated to some extent, as evidenced by the slight decreases in the amounts of H_2_ and O_2_. The additional loading of Cr was found to inhibit the formation of water (with 98.6% of the original gas mixture remaining). In contrast, if Cr_2_O_3_ was not loaded or only loaded in a small amount, the quantities of H_2_ and O_2_ gradually decreased over time. This result further demonstrated that the Cr_2_O_3_ layer was able to coat the Rh particles to prevent the backward reaction during the OWS process.

## Conclusions

A Z-scheme photocatalyst sheet incorporating Cr_2_O_3_/Rh/Ga-LTCA and CoO_*x*_/ITO/BVO serving as the HEP and OEP, respectively, was constructed using CNTs as the solid electron mediator, along with a filter paper substrate. This Cr_2_O_3_/Rh/Ga-LTCA-CNT-CoO_*x*_/ITO/BVO sheet system was able to split water into gaseous H_2_ and O_2_ stoichiometrically *via* a Z-scheme mechanism in response to visible light. An STH value of 0.17% was obtained at 313 K and 10 kPa, meaning that this sheet outperformed other systems as a result of surface modifications and optimized fabrication.^42,46^ Following two-step deposition of Cr_2_O_3_ onto the Ga-LTCA, the sheet system demonstrated prolonged photostability and Cr_2_O_3_ deposited using a specific process was demonstrated to block the reverse reaction, especially at high background pressures of 60 kPa and more. The optimized loading of ITO NPs effectively enhanced the performances of the BVO and so promoted the Z-scheme OWS activity, with an enhancement of 128%. This study demonstrates the application of a narrow-bandgap oxysulfide photocatalyst having an absorption edge close to 700 nm, superior to the majority of OWS systems reported to date in terms of harvesting visible light. The present work shows the rational design of a photocatalyst sheet system for Z-scheme OWS, employing a cost-effective carbon-based material as the conductive mediator. This technology could be scaled-up to allow practical solar-to-hydrogen conversion.

## Author contributions

L. Wang, T. Takata, and K. Domen conceived and designed the experiments. L. Wang. performed most characterizations and photocatalytic experiments. N. Zettsu produced the CNTs used in the experiments. L. Wang, C. Gu, and J. Yoshimura investigated the effect of ITO loading. C. Gu, S. Karade, and S. Nandy provided valuable suggestions with regard to the manuscript. L. Wang and T. Hisatomi. wrote the manuscript with contributions from the other co-authors. Y. Nishi and K. Kanie provided the ITO NPs used in the experiments. K. Domen supervised the research. The manuscript was written through contributions of all authors. All authors have given approval to the final version of the manuscript.

## Conflicts of interest

J. Y., Y. N., K. K., T. H., and K. D. have applied for a patent related to this work (Japanese Unexamined Patent Application Publication no. 2025-116676).

## Supplementary Material

SC-OLF-D5SC05277G-s001

## Data Availability

Additional data are available from the corresponding author upon reasonable request. All data supporting this study are available in the SI, including synthesis of photocatalyst, characterizations, and supplimentary photocatalytic performance tests. See DOI: https://doi.org/10.1039/d5sc05277g.
