# Supplementary material for: Z-scheme overall water splitting on photocatalyst sheet mediated by carbon nanotubes using oxysulfide photocatalyst responsive to long wavelengths
Source: Chem Sci. 2025 Sep 23;16(42):19720–6. doi: 10.1039/d5sc05277g (PMC12478603; doi:10.1039/d5sc05277g)
Supplement: SC-016-D5SC05277G-s001 [file SC-016-D5SC05277G-s001.pdf]

Supplementary Information (SI) for Chemical Science.
This journal is © The Royal Society of Chemistry 2025

Supporting Information

Z-scheme overall water splitting on photocatalyst sheet mediated by carbon nanotubes using oxysulphide photocatalyst responsive to long wavelengths

Long Wang,[ab] Chen Gu,[a] Takata Tsuyoshi,[c] Nobuyuki Zettsu,[a] Swapnil S. Karade,[a] Swarnava Nandy,[a] Joji Yoshimura,[a] Yasutaka Nishi,[d] Kiyoshi Kanie,[e] Takashi Hisatomi,[a] and Kazunari Domen*[af]

[a]Institute for Aqua Regeneration, Shinshu University, Wakasato 4-17-1, Nagano-shi, Nagano 380-8553, Japan.

[b]School of Materials and Energy, Guangdong University of Technology, Guangzhou 510006, China.

[c]Research Initiative for Supra-Materials, Shinshu University, Wakasato 4-17-1, Nagano-shi, Nagano 380-8553, Japan.

[d]Nikon Corporation, 10-1, Asamizodai, 1-chome, Minami-ku, Sagamihara-City 252-0328, Japan.

[e]International Center for Synchrotron Radiation Innovation Smart, Tohoku University, Sendai, Miyagi 980- 8577, Japan.

[f]Office of University Professors, The University of Tokyo, 2-11-16 Yayoi, Bunkyo-ku, Tokyo 113-8656, Japan.

Contents

1. Experimental sections ... 4

Fig. S1 (a) XRD patterns and (b) Ga 3d XPS spectra obtained from Ga-LTCA and LTCA. ... 8

Fig. S2 SEM images of (a) Ga-LTCA and (b) LTCA. ... 9

Fig. S3 XRD pattern for BVO prepared using hydrothermal method. ... 10

Fig. S4 Hydrogen evolution activity of Ga-LTCA as a function of (a) mass of Rh loaded by impregnation-reduction and (b) H_2 evolution over time using Ga-LTCA after loading with 0.4wt% Rh by different methods; Reaction conditions: 200 mg Rh/Ga-LTCA, 100 mL water containing 20 mM Na_2S/Na_2SO_3, under Ar at 5 kPa, Xe lamp with a 420 nm filter (L42). ... 11

Fig. S5 Hydrogen evolution activity of Ga-LTCA specimens loaded with different cocatalysts using an impregnation-reduction method. Reaction condition: 200 mg cocatalyst loaded Ga-LTCA, 100 mL water containing 20 mM Na_2S/Na_2SO_3, under Ar of 5 kPa, Xe lamp with a 420 nm filter (L42). ... 12

Fig. S6 Photocatalytic H_2 performance data for Ga-LTCA samples. (a) H_2 evolution over time using varying amounts of Cr_2O_3/Rh/Ga-LTCA in aqueous suspension containing 20 mM Na_2S/Na_2SO_3. (b) AQY data for Cr_2O_3/Rh/Ga-LTCA at different wavelengths together with the DRS spectrum of the material. Reaction conditions: 100 mL water containing 20 mM Na_2S/Na_2SO_3, 5 kPa of Ar, Xe lamp with a 420 nm filter (L42). ... 13

Fig. S7 Photocatalytic O_2 evolution performance for pristine and modified BVO samples. (a) O_2 evolution rates over varying amounts of BVO and CoO_x/BVO specimens in suspension containing $AgNO_3$ as a sacrificial reagent, and (b) AQY values for200 mg of CoO_x/BVO at different wavelengths and along with DRS data. Reaction condition: 100 mL water containing 20 mM $AgNO_3$ as sacrificial reagent, under Ar at 5 kPa, Xe lamp with a 420 nm filter (L42). ... 14

Fig. S8 OWS activity of Ga-LTCA/CNT/BVO sheets with various pre-loading amounts of Cr on Rh/Ga-LTCA. Reaction conditions: 15 and 45 mg Ga-LTCA and BVO, respectively, 1.0 wt% CNTs with respect to mass of BVO, 40 mL water, under Ar at 5 kPa, Xe lamp with a 420 nm filter (L42), no stirring. ... 15

Fig. S9 XPS spectra of (a) Cr 2p and (b) Ti 2p signals acquired for Cr_2O_3/Rh/Ga-LTCA before and after sheet fabrication. ... 16

Fig. S10 XPS spectra of (a) Rh 3d, (b) Cr 2p in 0.5 wt% Cr_2O_3/0.4 wt% Rh/Ga-LTCA specimen, and (c) the Co 2p XPS data obtained from a 0.5wt% CoO_x/BVO sample ... 17

Fig. S11 (a) OWS activity of Ga-LTCA/CNT/BVO sheets (as reflected by gas evolution rates) having (a) various CNT loadings relative to the mass of BVO and (b) different masses of Ga-LTCA and BVO (maintaining 1:3 ratio by mass). ... 18

Fig. S12 Photographic images of photocatalyst sheet prepared on filter paper using various masses of Ga-LTCA and BVO with constant 1:3 ratio. 19

Fig. S13 Amount of evolved gas evolution as function of time during the Z-scheme OWS using Ga-LTCA/CNT/BVO sheets with additional Cr loadings of (a) 0.25, (b) 0.5, and (c) 0.75 wt%., respectively, relative to mass of of Ga-LTCA. Reaction conditions: 15 and 45 mg Ga-LTCA and BVO, respectively, 1.0 wt% CNTs with respect to mass of BVO, 40 mL water, under Ar at 5 kPa, Xe lamp with a 420 nm filter (L42), no stirring. 20

Fig. S14 SEM images of pristine ITO, pure BVO, and BVO specimens loaded with varying amounts of ITO (values shown are the ITO percentages relative to the BVO mass). The scale bars each show 1 μm. 21

Fig. S15 XPS spectra of Sn 3d and In 3d collected from an ITO/BVO sample. 22

Fig. S16 Photocatalytic oxygen evolution reaction over BVO, CoO_x/BVO, and CoO_x/ITO/BVO specimens with ITO loaded using different techniques in latter case. Reaction conditions 100 mg catalyst, 100 mL water containing 20 mM $AgNO_3$ as a sacrificial reagent, under Ar at 5 kPa, Xe lamp with a 420 nm filter (L42). 23

Fig. S17 OWS activities of Ga-LTCA/CNT/BVO sheet as function of background pressure. Reaction conditions: 15 and 45 mg Ga-LTCA and BVO, respectively, 1.0 wt% CNTs with respect to the mass of BVO, 40 mL water, under Ar at pressures from 4 to 90 kPa, Xe lamp with a 420 nm filter (L42), no stirring. 24

Fig. S18 OWS performance of Ga-LTCA/CNT/BVO sheet prepared on filter paper, as reflected in volume of evolved H_2 and O_2. Reaction conditions: continuous water splitting using a panel system containing 15 mL of water under ambient pressure at room temperature with a Xe lamp having a 420 nm cutoff filter (L42) at a distance of 10 cm. 25

Table S1. Comparison of OER and AQY for BVO samples with different surface modifications.[a] 26

2. References......27

1. Experimental sections

1.1 Preparation of photocatalysts

Ga-doped $La_5Ti_2Cu_{0.9}Ag_{0.1}O_7S_5$ (designated herein as Ga-LTCA) having a Ga concentration of 1.0 mol% relative to the amount of Ti was synthesized through a solid-state reaction (SSR) approach.[1] High-purity starting materials, including La_2O_3 (99.99%, Kanto Chemical Co., Inc.), La_2S_3 (99.9%, High Purity Chemical Laboratory Co., Ltd.), TiO_2 (rutile phase, 99.99%, Kanto Chemical Co. Inc.), Ga_2O_3 (99.99%, High Purity Chemical Laboratory Co., Ltd.), Cu_2S (99%, High Purity Chemical Laboratory Co., Ltd.), Ag_2S (99%, High Purity Chemical Laboratory Co., Ltd.), and elemental S (99.99%, High Purity Chemical Laboratory Co., Ltd.), were precisely weighed according to the stoichiometric molar ratio of La_2O_3: La_2S_3: TiO_2: Ga_2O_3:Cu_2S:Ag_2S:S=2:3:3.96:0.02:0.9:0.1:0.5. All weighing and mixing procedures were conducted in a nitrogen-filled glove box to prevent the reagents from undergoing oxidation in air or asorbing moisture and carbon dioxide. Prior to mixing, La_2O_3 and TiO_2 were subjected to pre-calcination at 1273 K for 10 h and 1073 K for 1 h, respectively, to ensure complete dehydration and phase stabilization. The precursors were thoroughly ground and mixed to achieve homogeneity and sealed in an evacuated quartz tube. The thermal treatment protocol involved a controlled heating process: initially ramping from ambient temperature to 473 K over 9 min, followed by a gradual increase to 673 K over 100 min, and subsequently heating to the final synthesis temperature of 1323 K over 53 h. The sample was maintained at 1323 K for 96 h to ensure complete reaction and crystallization. After the thermal treatment, the material was allowed to cool naturally to room temperature, after which the resulting solid product was collected for characterization.

$BiVO_4$ (BVO) was prepared by a hydrothermal method in a previously reported literature.[2] Briefly, the precursors NH_4VO_3 (99.0%, Fujifilm Wako Pure Chemical Co.,) (10 mmol), $Bi(NO_3)_3{\cdot}5H_2O$ (99.5%, Fujifilm Wako Pure Chemical Co.,) (10 mmol) were added to 60 mL of a 2 M HNO_3 solution in a beaker. The pH of the solution was adjusted to 0.5 by the dropwise adding of an aqueous ammonia solution (25 wt%, Fujifilm Wako Pure Chemical Co.). After magnetically stirring for 2 h, the light-yellow precipitate in the beaker was transferred to a 100 mL Teflon-lined stainless-steel autoclave and hydrothermally treated at 473 K for 10 h in a temperature programmed oven. After the autoclave cooled to room temperature, the resulting bright yellow colored powder was collected by filtration and washed then dried at 313 K overnight.

Carbon nanotubes (CNTs) used in this study were provided by Meijo Nano Carbon Co., Ltd (EC2.0P, 0.2 wt%). The detailed preparation procedure can be found in the literature.[2]

1.2 Deposition of cocatalysts

Rh and Cr_2O_3 were loaded onto the Ga-LTCA by impregnation-reduction method and photodeposition method, respectively. Typically, a calculated amount of $RhCl_3 \cdot 3H_2O$ (~40%, Fujifilm Wako Pure Chemical Co.) was added into a Ga-LTCA photocatalyst suspension in water. After drying on a water-bath, the sample was put into a hydrogen gas furnace at 573K for 1h. A calculated amount of K_2CrO_4 was added in the solution containing Rh/Ga-LTCA photocatalyst. Then the suspension solution was irradiated with a Xe lamp (λ>300 nm) for 1 h . Note that the additional Cr were loaded after the photocatalyst sheet was fabricated by adding K_2CrO_4 was added to the solution and was allowed to irradiated for 1 h.

CoO_x and tin-doped indium oxide (ITO) was loaded onto the BVO by photodeposition and impregnation-reduction method, respectively. In this process, a quantity of BVO powder was dispersed in 150 mL of a potassium phosphate buffer solution (pH 6.0, 50 mM) containing $Co(NO_3)_2 \cdot 6H_2O$ (99.5%, Fujifilm Wako Pure Chemical Co) as the cobalt source, at various concentration of 0.5 wt% Co with respect to the amount of BVO. The resulting dispersion was irradiated with a 300 W Xe lamp with the full-arc for 1 h and then washed with distilled water and filtered to obtain the CoO_x/BVO. Regarding ITO loading, a quantity of BVO powder was dispersed in distilled water containing a calculated amount of ITO (3 wt%). After drying on a water-bath, the sample was put on a furnace flowed with pure nitrogen gas, hydrogen (5%) and nitrogen (95%) gases , an in air at 423-523 K for 1h. CoO_x/ITO/BVO was prepared by loading ITO and CoO_x step by step.

1.3 Preparation of photocatalyst sheets

The photocatalyst sheets were fabricated using a filtration method. In a typical procedure, predetermined quantities of Cr_2O_3/Rh/Ga-LTCA and CoO_x/$BiVO_4$ were dispersed in an aqueous solution and then subjected to ultrasonication for 5 minutes to ensure uniform dispersion. Subsequently, a measured amount of carbon nanotubes (CNTs) was introduced into the suspension, followed by continuous magnetic stirring to achieve a homogeneous mixture. The resulting suspension was then transferred to a Buchner funnel equipped with a 55 mm diameter filter paper for vacuum filtration. Following complete water removal, the filter paper containing the deposited photocatalyst composite was carefully retrieved and dried in an oven at a controlled temperature for 12 hours. To investigate the effect of

component ratios, four distinct weight ratios of Cr_2O_3/Rh/Ga-LTCA to $CoO_x/BiVO_4$ were prepared, maintaining a constant ratio of 1:3, specifically: 5 and 15, 10 and 30, 15 and 45, and 20 and 60 mg, respectively.

1.4 Photocatalytic water splitting reactions

Photocatalytic water splitting experiments were conducted using a closed gas circulation system equipped with a Pyrex top-irradiation vessel reactor. The fabricated photocatalyst sheet (55 mm in diameter, effective area: ~16 cm^2) was carefully positioned in the reactor containing 40 mL of distilled water. Prior to illumination, the system underwent multiple evacuation cycles to ensure complete removal of air. The photocatalytic reaction was initiated by irradiating the catalyst sheet with a 300 W Xenon lamp equipped with a 420 nm cutoff filter (L42). The system pressure was adjusted to 4 kPa or higher by introducing Ar gas, as required by experimental conditions. Evolved gases were quantitatively analyzed using a gas chromatography system (GC-8A, Shimadzu Corp.) featuring molecular sieve 5A columns and a thermal conductivity detector (TCD), with argon serving as the carrier gas. For solar-to-hydrogen (STH) energy conversion efficiency measurements values were determined by replacing the Xe lamp with an AM 1.5G solar simulator.

Half-reaction experiments, including the sacrificial hydrogen evolution reaction (HER) and oxygen evolution reaction (OER), were performed under identical system configurations and illumination conditions (λ > 420 nm). For the HER measurements, varying quantities of HEP were employed in conjunction with an aqueous solution (100 mL) containing 20 mM Na_2S and 20 mM Na_2SO_3 as electron donors at a system pressure of 8 kPa. In photocatalytic OER experiments, $BiVO_4$ modified with varying amounts of CoO_x were dispersed in 100 mL of an aqueous solution containing 10 mM $AgNO_3$ as an electron scavenger.

1.5 Characterization

Powdered X-ray diffraction (XRD) analyses were performed using a Rigaku MiniFlex 300 powder diffractometer. Field-emission scanning electron microscopy (FE-SEM) was carried out on Hitachi SU8000 and Phenom Pharos Desktop SEM, Thermo Fisher Scientific. SEM EDS mapping was recorded on with energy dispersive X-ray spectroscopy (SEM EDS, JSM-7800F, JEOL). UV-vis diffuse reflectance spectra (DRS) were conducted on a spectrophotometer (V-670, JASCO) with an integrating sphere. X-ray photoelectron spectroscopy (XPS) analyses were carried out with a PHI Quantera II instrument (ULVAC-PHI, Inc.) employing a monochromatized Al Kα line source.

1.6 Apparent quantum yield assessments

The Apparent quantum yield (AQY) experiments were calculated as:

$$\mathrm{AQY}\ (\%) = \frac{N_e}{N_p} \times 100\% = \frac{A\, n_{H2,t} hc}{PS\lambda_{inc} t} \times 100\% \qquad (1)$$

where N_e is the quantity of electrons involved in the reaction, N_p is the incident photons, $n_{H_{2,t}}$ is the amount of hydrogen evolution, *h* is Planck's constant, *c* is the speed of light in vacuum, *P* is the power density of the incident monochromatic light, *S* is the irradiation area, λ_{inc} is the wavelength of the incident monochromatic light, and *t* is the duration of incident light exposure. For the half reaction of H_2 and O_2, the value of A, the coefficient representing the number of electrons involved in the redox reaction, is 2 and 4, respectively. The value of S_{area} was adjusted to 9 cm^2 in the present work.

1.7 Solar-to-hydrogen energy conversion efficiency measurements

The water splitting reaction trials were carried out under simulated sunlight (AM 1.5G) and the solar-to-hydrogen (STH) energy conversion efficiency values were calculated as formula 2:

$$STH\ (\%) = \frac{R_{H_2} \times \Delta G_r}{P \times S} \times 100\%, \qquad (2)$$

where $R(H_2)$, ΔG_r, *P* and *S* represent the hydrogen evolution rate during the overall water splitting reaction, the Gibbs energy change for the reaction $H_2O(l) \rightarrow H_2(g) + 1/2O_2(g)$, the energy intensity of the AM 1.5G solar irradiation (XES-40S1, San-EI Electric) at an intensity of 100 mW/cm^2 and the irradiation area (9 cm^2), respectively. The ΔG_r value used for the calculations was 237 kJ mol^{-1}.

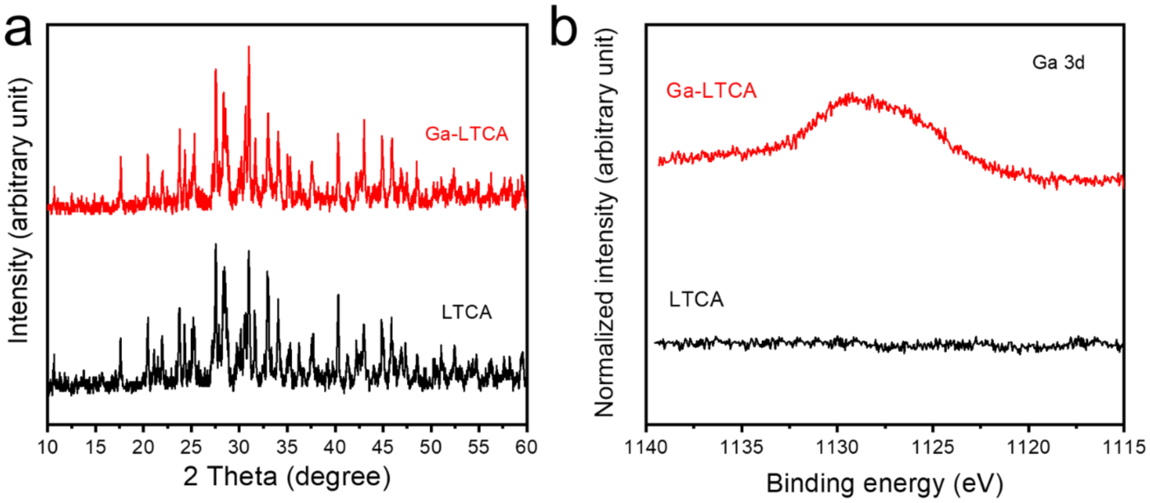

Fig. S1 (a) XRD patterns and (b) Ga 3d XPS spectra obtained from Ga-LTCA and LTCA.

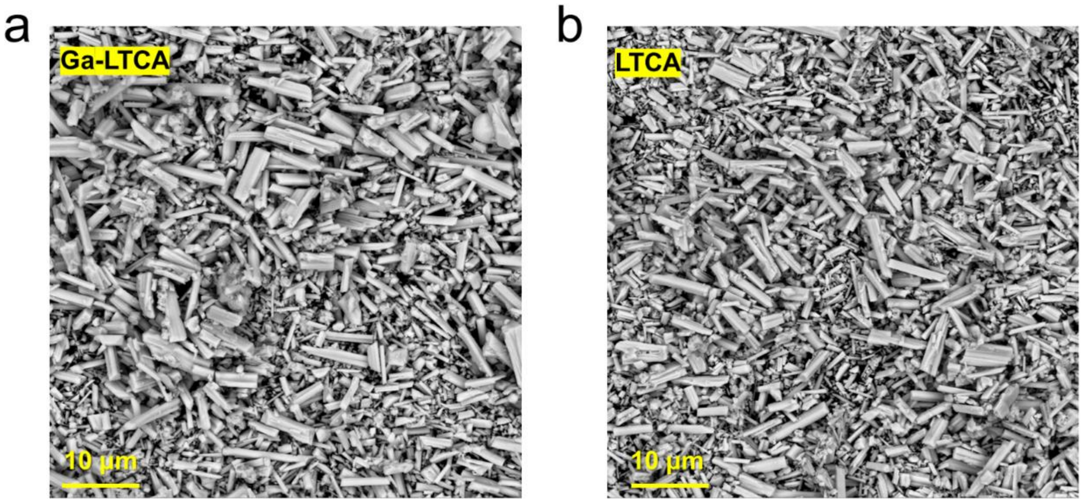

Fig. S2 SEM images of (a) Ga-LTCA and (b) LTCA.

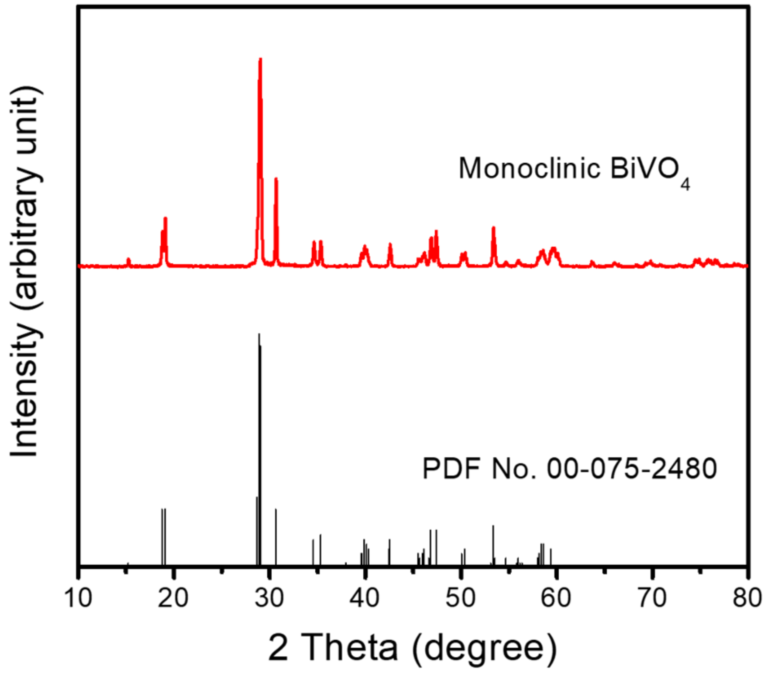

Fig. S3 XRD pattern for BVO prepared using hydrothermal method.

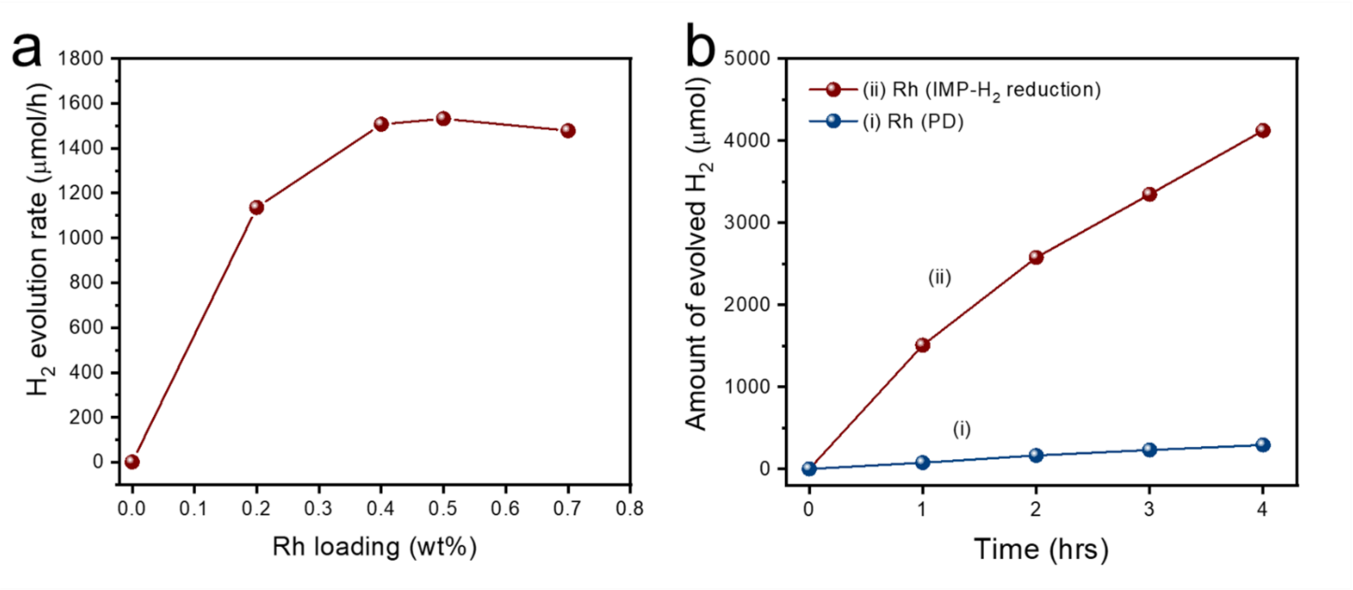

Fig. S4 Hydrogen evolution activity of Ga-LTCA as a function of (a) mass of Rh loaded by impregnation-reduction and (b) H_2 evolution over time using Ga-LTCA after loading with 0.4wt% Rh by different methods; Reaction conditions: 200 mg Rh/Ga-LTCA, 100 mL water containing 20 mM Na_2S/Na_2SO_3, under Ar at 5 kPa, Xe lamp with a 420 nm filter (L42).

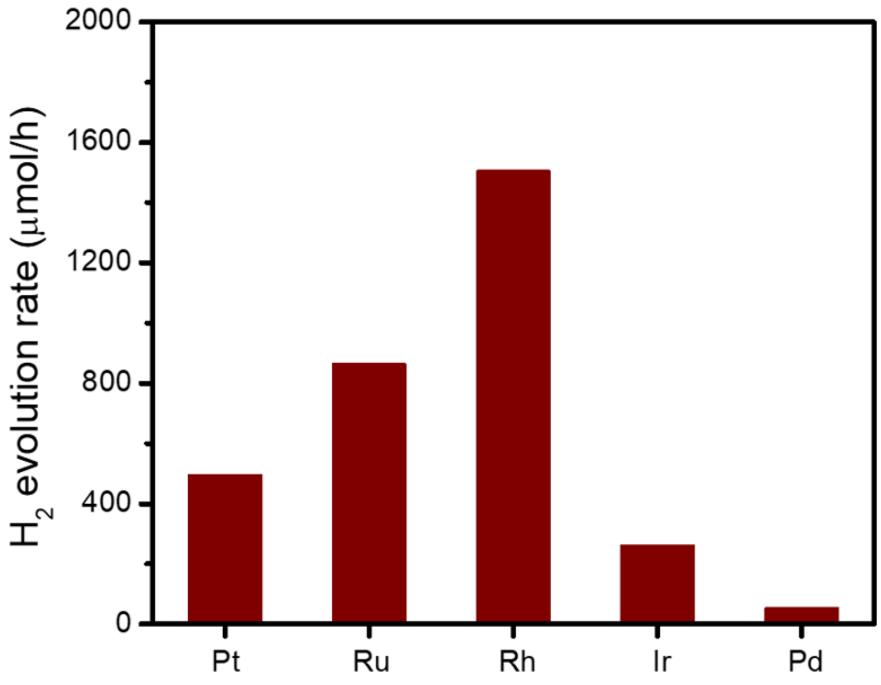

Fig. S5 Hydrogen evolution activity of Ga-LTCA specimens loaded with different cocatalysts using an impregnation-reduction method. Reaction condition: 200 mg cocatalyst loaded Ga-LTCA, 100 mL water containing 20 mM Na_2S/Na_2SO_3, under Ar of 5 kPa, Xe lamp with a 420 nm filter (L42).

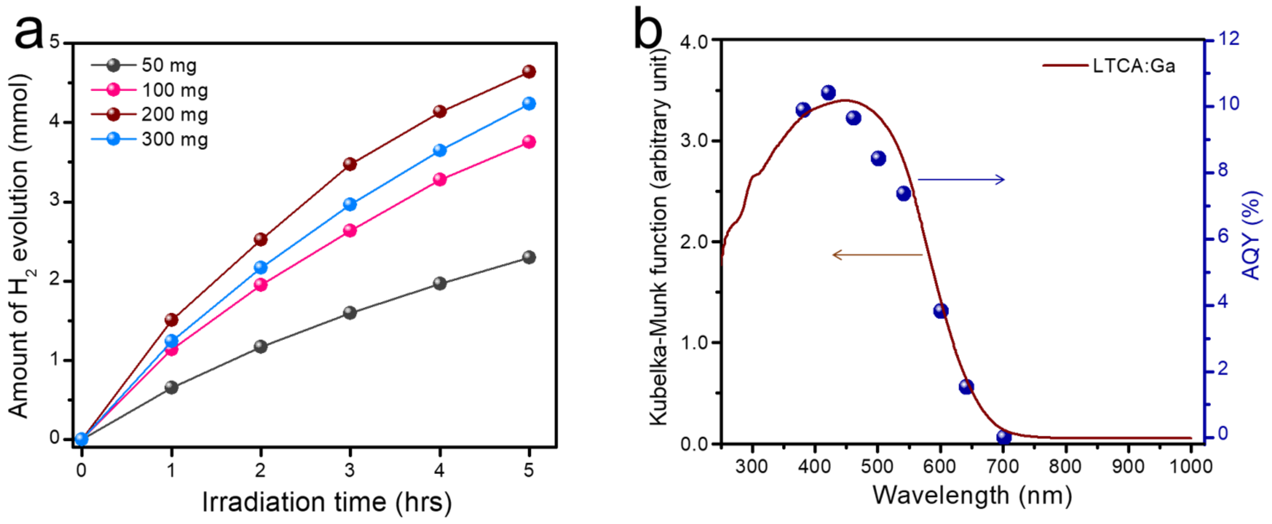

Fig. S6 Photocatalytic H_2 performance data for Ga-LTCA samples. (a) H_2 evolution over time using varying amounts of Cr_2O_3/Rh/Ga-LTCA in aqueous suspension containing 20 mM Na_2S/Na_2SO_3. (b) AQY data for Cr_2O_3/Rh/Ga-LTCA at different wavelengths together with the DRS spectrum of the material. Reaction conditions: 100 mL water containing 20 mM Na_2S/Na_2SO_3, 5 kPa of Ar, Xe lamp with a 420 nm filter (L42).

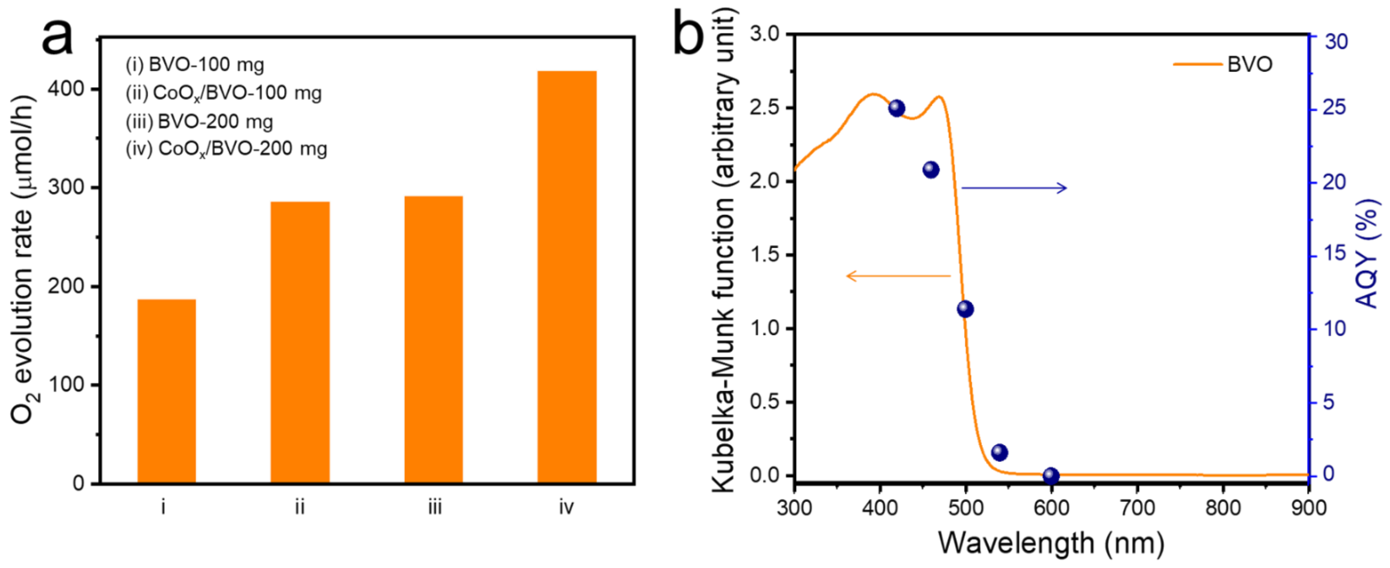

Fig. S7 Photocatalytic O_2 evolution performance for pristine and modified BVO samples. (a) O_2 evolution rates over varying amounts of BVO and CoO_x/BVO specimens in suspension containing $AgNO_3$ as a sacrificial reagent, and (b) AQY values for200 mg of CoO_x/BVO at different wavelengths and along with DRS data. Reaction condition: 100 mL water containing 20 mM $AgNO_3$ as sacrificial reagent, under Ar at 5 kPa, Xe lamp with a 420 nm filter (L42).

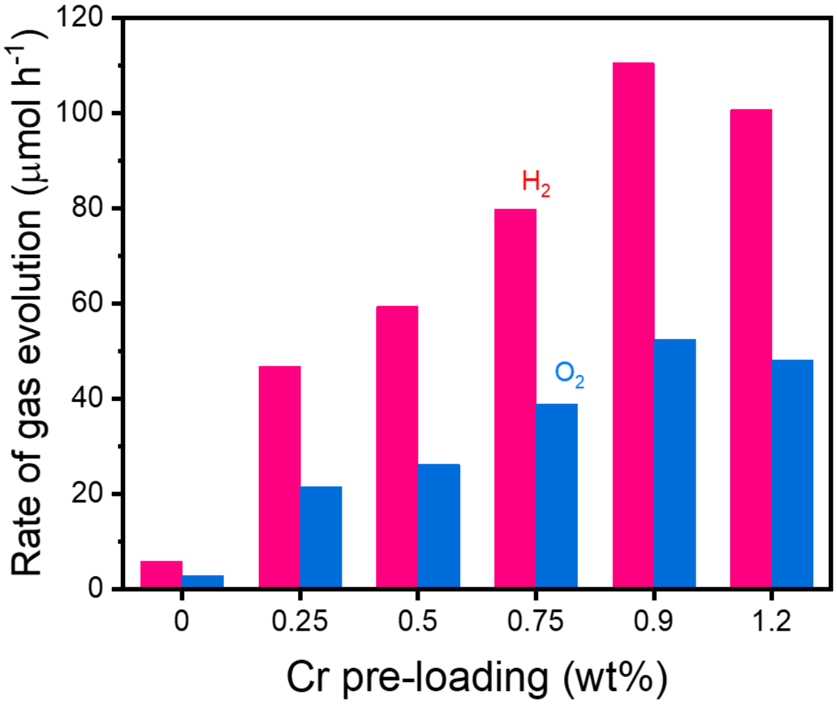

Fig. S8 OWS activity of Ga-LTCA/CNT/BVO sheets with various pre-loading amounts of Cr on Rh/Ga-LTCA. Reaction conditions: 15 and 45 mg Ga-LTCA and BVO, respectively, 1.0 wt% CNTs with respect to mass of BVO, 40 mL water, under Ar at 5 kPa, Xe lamp with a 420 nm filter (L42), no stirring.

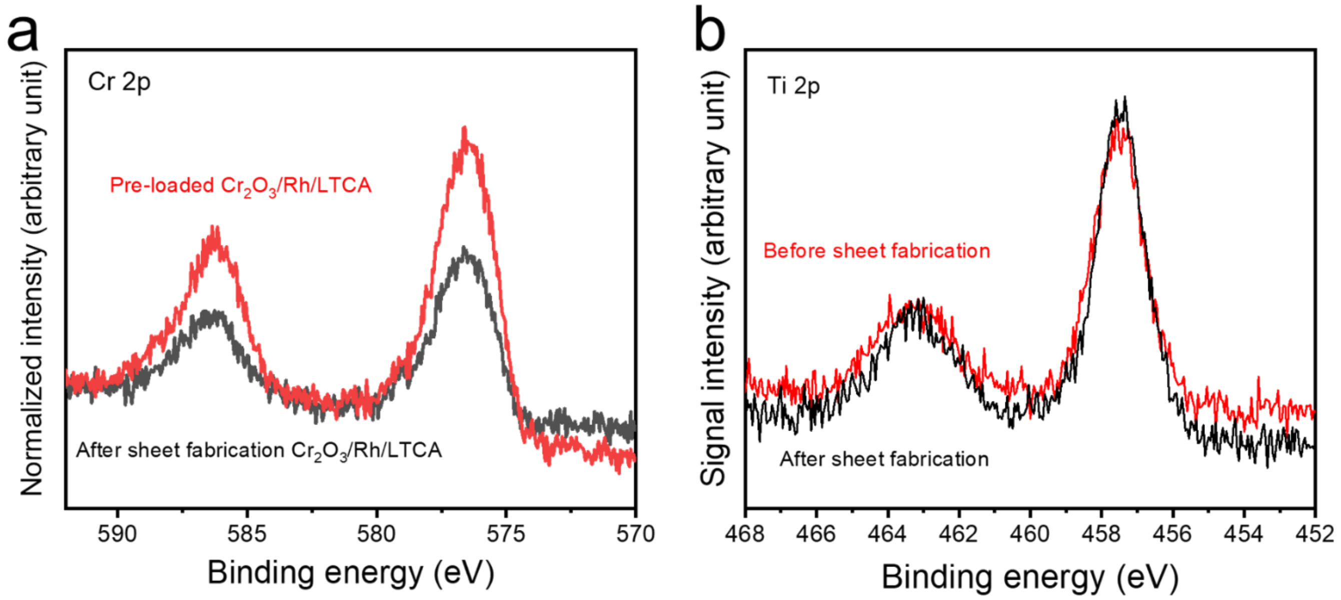

Fig. S9 XPS spectra of (a) Cr 2p and (b) Ti 2p signals acquired for Cr_2O_3/Rh/Ga-LTCA before and after sheet fabrication.

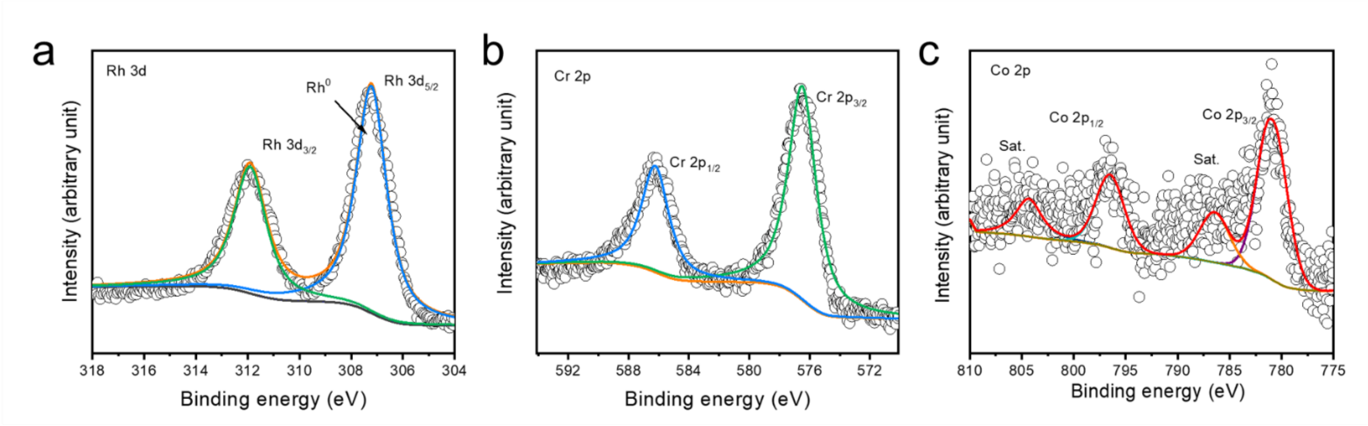

Fig. S10 XPS spectra of (a) Rh 3d, (b) Cr 2p in 0.5 wt% Cr_2O_3/0.4 wt% Rh/Ga-LTCA specimen, and (c) the Co 2p XPS data obtained from a 0.5wt% CoO_x/BVO sample.

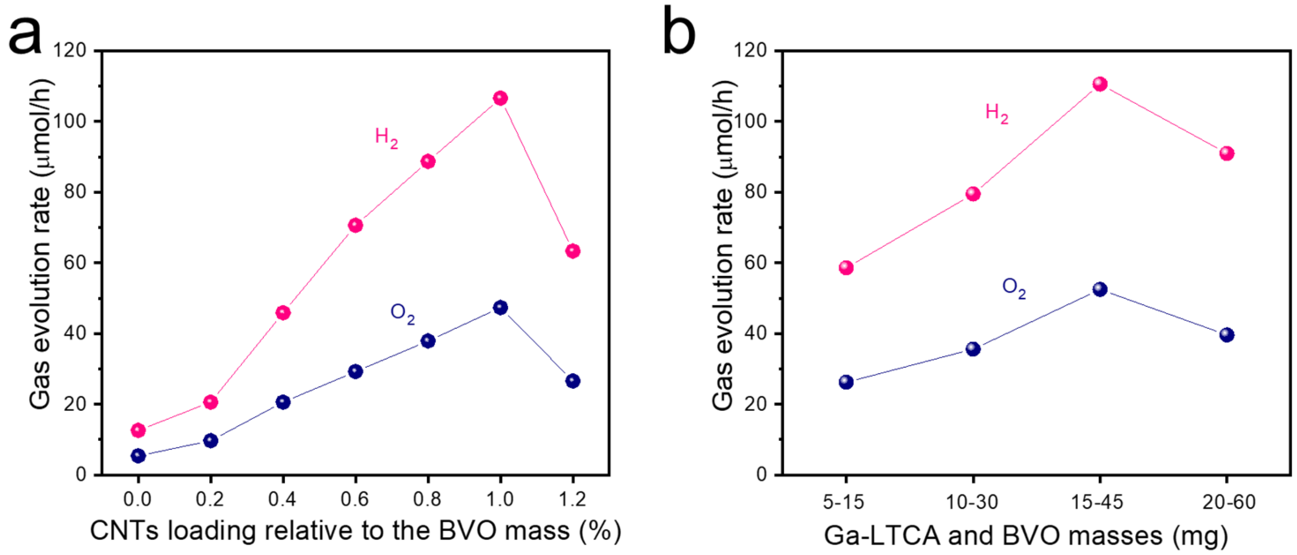

Fig. S11 (a) OWS activity of Ga-LTCA/CNT/BVO sheets (as reflected by gas evolution rates) having (a) various CNT loadings relative to the mass of BVO and (b) different masses of Ga-LTCA and BVO (maintaining 1:3 ratio by mass).

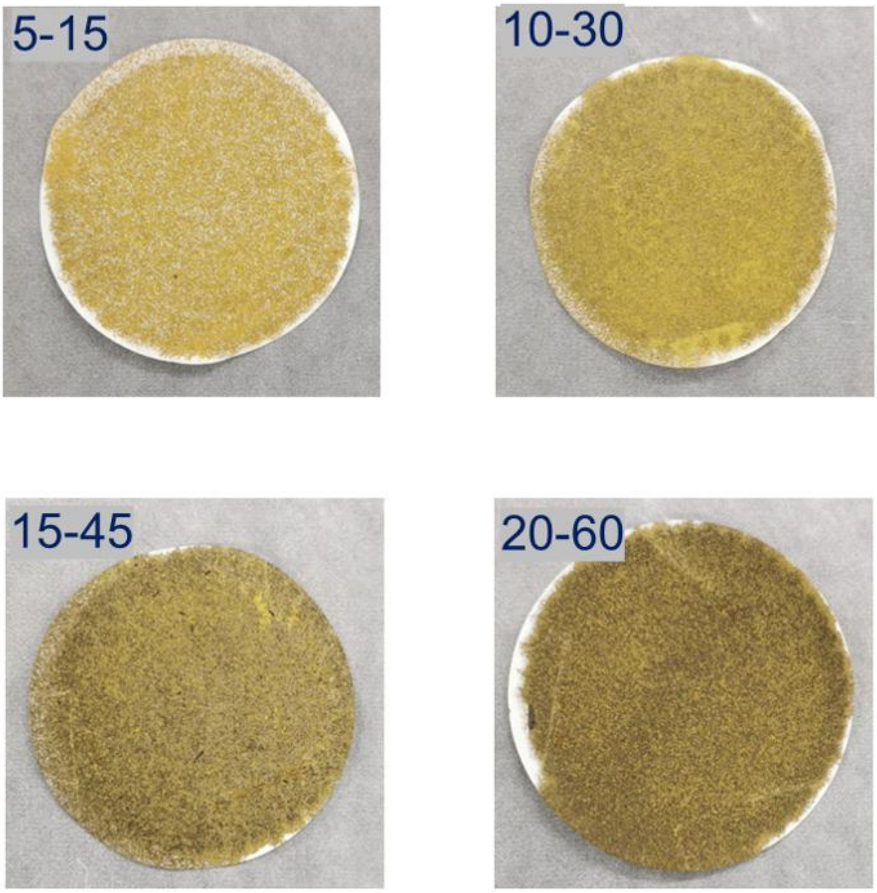

Fig. S12 Photographic images of photocatalyst sheet prepared on filter paper using various masses of Ga-LTCA and BVO with constant 1:3 ratio.

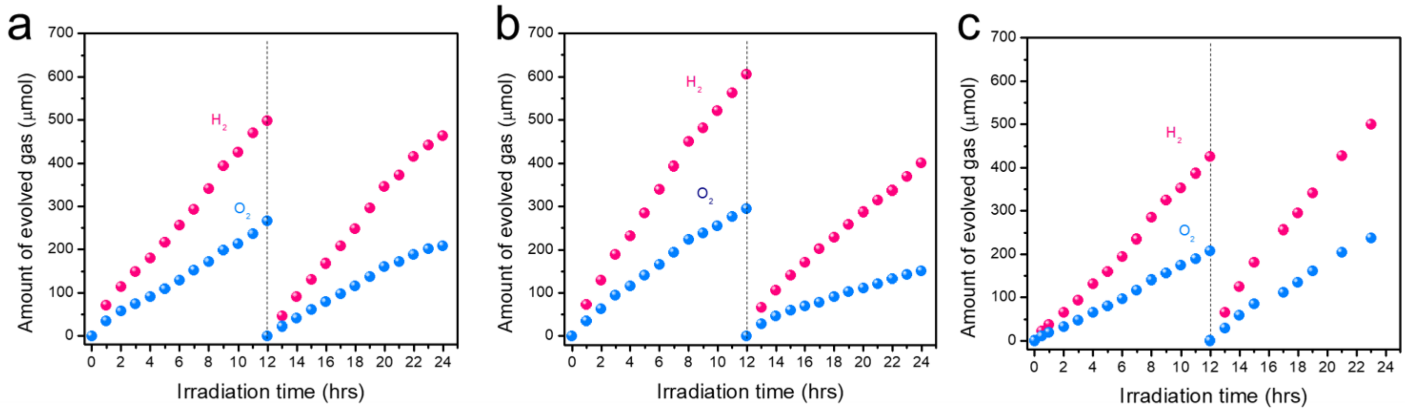

Fig. S13 Amount of evolved gas evolution as function of time during the Z-scheme OWS using Ga-LTCA/CNT/BVO sheets with additional Cr loadings of (a) 0.25, (b) 0.5, and (c) 0.75 wt%., respectively, relative to mass of of Ga-LTCA. Reaction conditions: 15 and 45 mg Ga-LTCA and BVO, respectively, 1.0 wt% CNTs with respect to mass of BVO, 40 mL water, under Ar at 5 kPa, Xe lamp with a 420 nm filter (L42), no stirring.

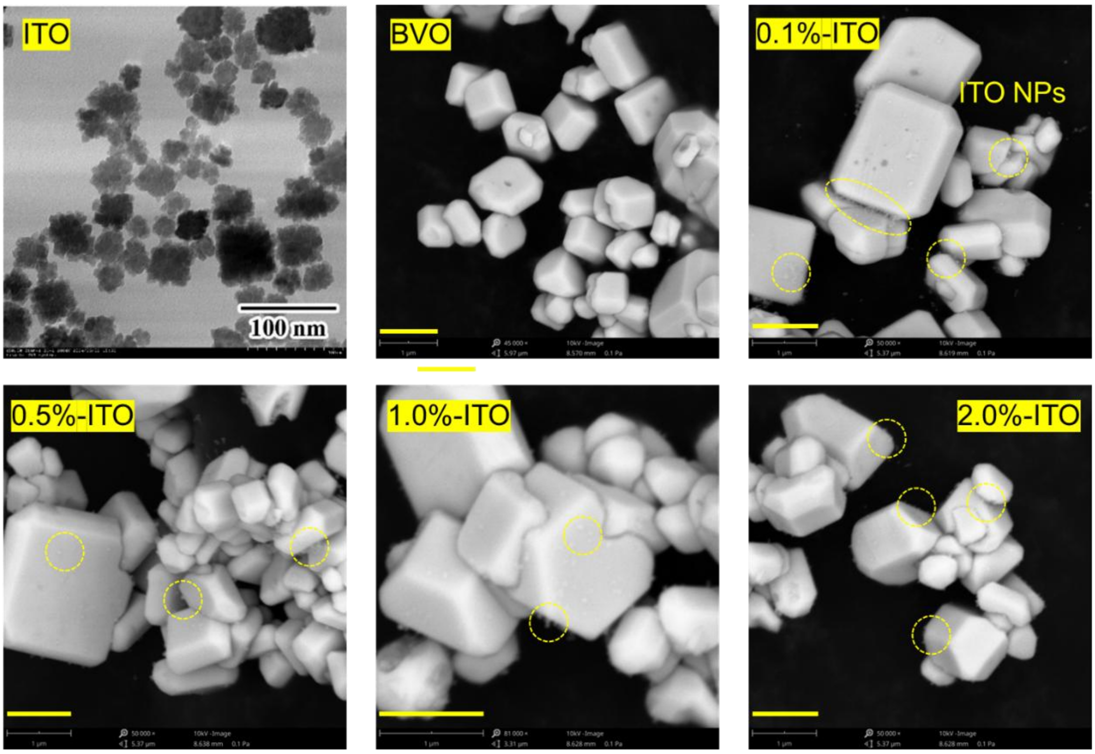

Fig. S14 SEM images of pristine ITO, pure BVO, and BVO specimens loaded with varying amounts of ITO (values shown are the ITO percentages relative to the BVO mass). The scale bars each show 1 µm.

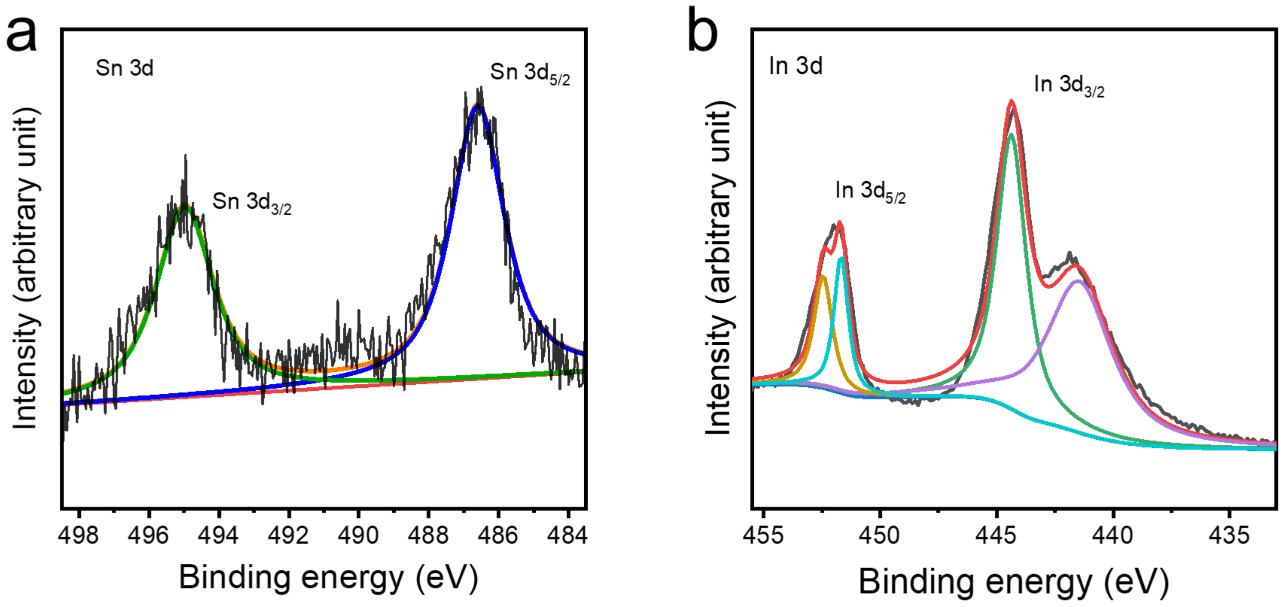

Fig. S15 XPS spectra of Sn 3d and In 3d collected from an ITO/BVO sample.

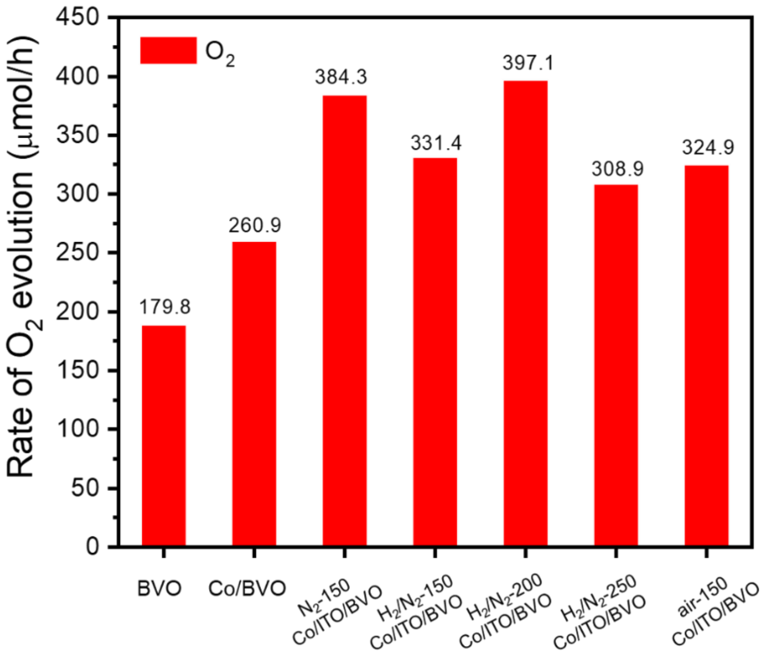

Fig. S16 Photocatalytic oxygen evolution reaction over BVO, CoO_x/BVO, and CoO_x/ITO/BVO specimens with ITO loaded using different techniques in latter case. Reaction conditions 100 mg catalyst, 100 mL water containing 20 mM $AgNO_3$ as a sacrificial reagent, under Ar at 5 kPa, Xe lamp with a 420 nm filter (L42).

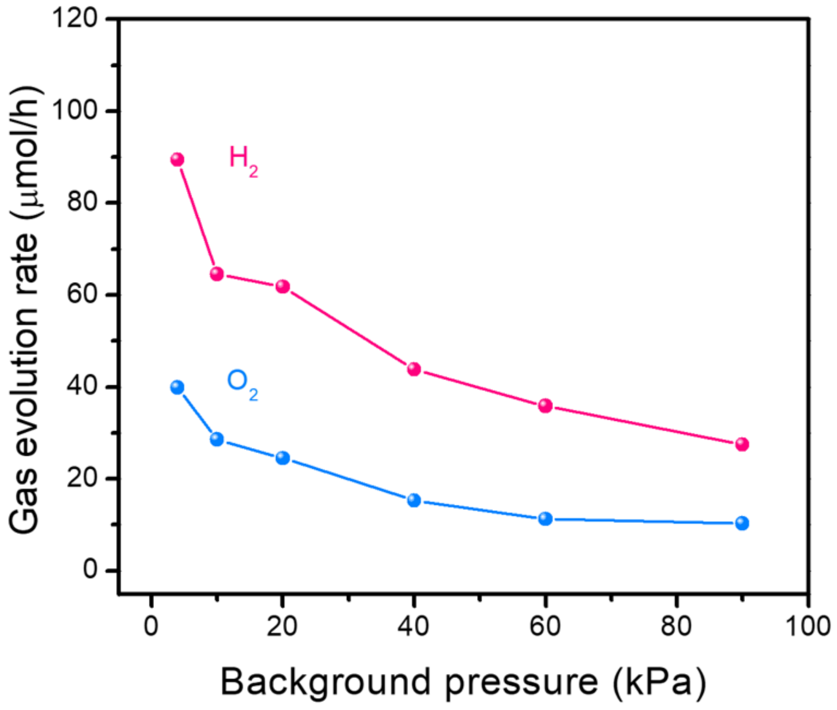

Fig. S17 OWS activities of Ga-LTCA/CNT/BVO sheet as function of background pressure. Reaction conditions: 15 and 45 mg Ga-LTCA and BVO, respectively, 1.0 wt% CNTs with respect to the mass of BVO, 40 mL water, under Ar at pressures from 4 to 90 kPa, Xe lamp with a 420 nm filter (L42), no stirring.

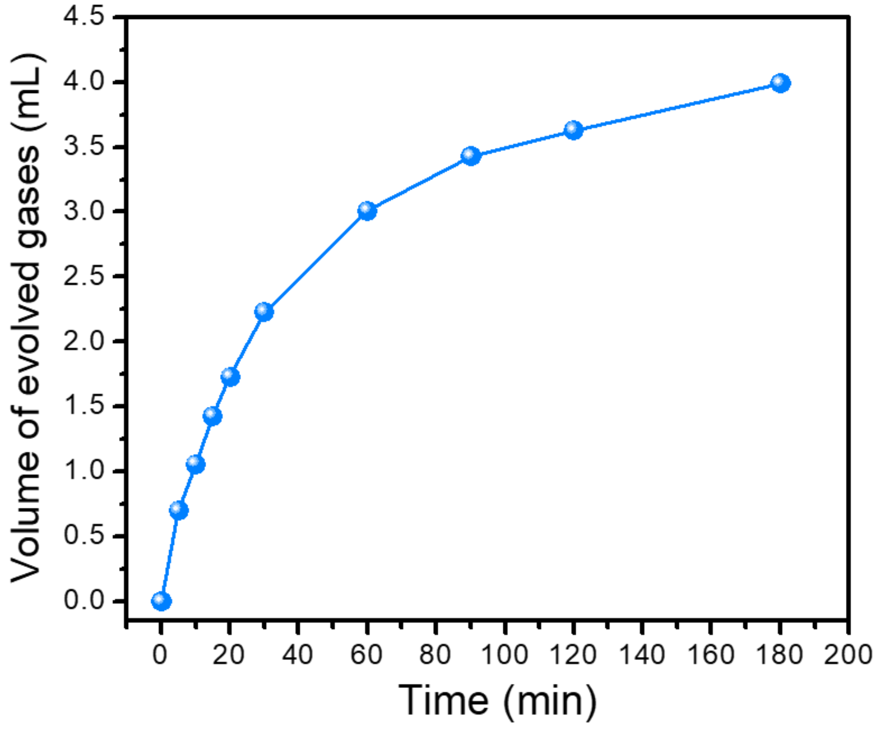

Fig. S18 OWS performance of Ga-LTCA/CNT/BVO sheet prepared on filter paper, as reflected in volume of evolved H_2 and O_2. Reaction conditions: continuous water splitting using a panel system containing 15 mL of water under ambient pressure at room temperature with a Xe lamp having a 420 nm cutoff filter (L42) at a distance of 10 cm.

Table S1. Comparison of OER and AQY for BVO samples with different surface modifications.[a]

Sample	OER rate (μmol h^{-1})[b]	AQY (%)[c]
BVO	180	16
CoO_x/BVO	260	25
CoO_x/ITO/BVO	398	36

[a]Reaction conditions: 100 mg catalyst, 100 mL water containing 20 mM $AgNO_3$ as a sacrificial reagent, under Ar at 5 kPa.

[b]Measured under illumination from a Xe lamp with a cut-off filter (λ > 420 nm)

[c]Measured under illumination from a Xe lamp with a band pass filter (λ = 420 nm).

2. References

1. C. Gu, Y. Miseki, H. Nishiyama, T. Takata, J. Yoshimura, Y. Ma, L. Lin, T. Hisatomi, D. Lu, N. Zettsu, Y. Nishina and K. Domen, *Chem Catal*, 2025, **5**, 101233.
2. L. Lin, Y. Ma, N. Zettsu, J. J. M. Vequizo, C. Gu, A. Yamakata, T. Hisatomi, T. Takata and K. Domen, *J. Am. Chem. Soc.*, 2024, **146**, 14829-14834.
